# Post-COVID-19 physical and cognitive impairments and associations with quality of life: a cross-sectional study

**DOI:** 10.3389/fspor.2024.1246585

**Published:** 2024-03-05

**Authors:** Fatemeh Rahimi, Maryam Saadat, Masumeh Hessam, Majid Ravanbakhsh, Saeideh Monjezi

**Affiliations:** ^1^ Student Research Committee, Ahvaz Jundishapur University of Medical Sciences, Ahvaz, Iran; ^2^ Rehabilitation Research Center, Ahvaz Jundishapur University of Medical Sciences, Ahvaz, Iran; ^3^ Department of Physiotherapy, School of Rehabilitation Sciences, Ahvaz Jundishapur University of Medical Sciences, Ahvaz, Iran

**Keywords:** COVID-19, long COVID, physical impairment, cognitive impairment, quality of life

## Abstract

**Background and objective:**

This study aimed to compare physical and cognitive functions between post-coronavirus disease 2019 (COVID-19) participants and healthy matched controls and investigate associations between physical and cognitive impairments with quality of life.

**Methods:**

Twenty-three post-COVID-19 participants and 23 age and sex-matched healthy people without a history of COVID-19 were included. Physical function was assessed using the Medical Research Council Sum Score (MRC-SS), 2 min Step Test, Modified Borg Scale, and Short Physical Performance Battery (SPPB) Test. Cognitive function was assessed using the Montreal Cognitive Assessment (MoCA), Trail Making Test (TMT), and Stroop test, and the quality of life was evaluated using the Euro Quality of Life-5 Dimensions-3 Levels (EQ-5D-3l) questionnaire. Assessments were performed by a physical therapist in one session.

**Results:**

Mann-Whitney *U* test showed that in the post-COVID-19 group, compared to the control group, the number of steps in the 2 min Step Test (*p *< 0.001, ES = 0.57) and the scores of the SPPB (*p *= 0.03, ES = 0.32), MoCA (*p *= 0.003, ES = 0.44), Stroop test (*p *< 0.001, ES = 0.75), and the EQ-5D-3l visual analog scale (*p *= 0.027, ES = 0.32) were significantly lower. In addition, the Modified Borg Scale score (*p *< 0.001, ES = 0.6), TMT-A (*p *= 0.013, ES = 0.36) and TMT-B (*p *= 0.016, ES = 0.35) times, and the Stroop time (*p *< 0.001, ES = 0.61) were significantly higher in the post-COVID-19 group. There were no significant between-group differences in the MRC-SS score (*p *= 0.055, ES = 0.28). Furthermore, there were significant moderate to high associations between physical and cognitive functions and the quality of life in post-COVID-19 participants.

**Conclusions:**

On average 4 months after symptomatic COVID-19, post-COVID-19 participants had significant impairments in physical and cognitive functions compared to healthy matched controls that were significantly correlated with the quality of life. These findings highlight the need for a comprehensive assessment to plan appropriate management strategies.

## 1 Introduction

A significant proportion of people infected with Coronavirus disease 2019 (COVID-19) can experience symptoms that last beyond the initial illness (1, 2). According to the National Institute of Health and Care Excellence (NICE), symptoms persisting longer than 12 weeks following the acute onset of COVID-19 are defined as “long COVID” or “post-COVID-19 syndrome” (3). Post-COVID-19 syndrome can occur in mild-intensity COVID-19 patients of all ages who do not have any pre-existing chronic disease (2, 4). The estimated incidence in non-hospitalized patients is 10%–30%, and in hospitalized patients is 50%–70% (5).

The most common symptoms of post-COVID-19 syndrome are fatigue, pain, muscle weakness, dyspnea, disturbed sleep, and cognitive and mental impairments (2, 6, 7). Complex mechanisms are involved in physical and cognitive impairments in post-COVID-19 syndrome. Studies imply neuro-inflammation, immune dysregulation, auto-immunity, endothelial abnormalities, and coagulation activation as the main underlying pathophysiological mechanisms (5, 8). The physical and cognitive systems can influence each other's function (9, 10). A recent study showed a significant correlation between physical function (measured by the 6 min walk test, 30 s sit-to-stand test, and handgrip strength) and cognitive function (measured by the Screen for Cognitive Impairment in Psychiatry-Danish version (SCIP-D) and Trail Making Test-Part B (TMT-B)) in post-COVID-19 individuals that can denote common mechanisms triggering both physical and cognitive impairments (7).

Persistent symptoms associated with post-COVID-19 syndrome (e.g., fatigue, brain fog, muscle weakness) can affect participation in social roles and reduce health-related quality of life, which is a principal indicator of the impact of diseases on physical, psychological, and social domains (4, 11). Health-related quality of life is also an important factor to identify patients with a high burden of post-COVID-19 syndrome (12). Therefore, comprehensive knowledge of the affected physical and cognitive functions in post-COVID-19 individuals and their association with quality of life is crucial to guide more appropriate interventions. In spite of studies that have determined some aspects of physical and cognitive impairments in post-COVID-19 syndrome or compared these impairments between non-hospitalized and hospitalized patients, there are limited studies comparing physical and cognitive impairments and their association with quality of life between post-COVID-19 individuals and healthy matched controls. Recently, Miskowiak et al. reported moderate to large impairments in global cognition and working memory and mild to moderate impairments in verbal fluency, verbal learning, and delayed verbal memory among patients in a long-COVID clinic (on average 7 months after acute COVID) compared to healthy controls (13).

The main purpose of the present study was to compare physical and cognitive functions between post-COVID-19 individuals without preexisting locomotor disabilities and healthy matched controls. The secondary aim was to investigate associations between physical and cognitive impairments with quality of life.

## 2 Methods

### 2.1 Study design and setting

This cross-sectional study was conducted at the Rehabilitation Research Center, Ahvaz Jundishapur University of Medical Sciences, Ahvaz, Iran, between May and November 2022. Ethics was approved by the Ethical Committee of Ahvaz Jundishapur University of Medical Sciences (IR.AJUMS.REC.1401.105).

### 2.2 Participants

People with post-COVID-19 were recruited and included if they had passed at least 3 months and maximum of 6 months since COVID-19 diagnosis (diagnosis based on the positive Polymerase Chain Reaction test) with mild to moderate disease severity and age between 18 and 65 years. Disease severity was determined based on the criteria for clinical severity of confirmed COVID-19 pneumonia. So, mild clinical symptoms and no imaging findings of pneumonia were considered as mild disease severity, whereas fever, respiratory symptoms, and imaging findings of pneumonia were considered as moderate disease severity (14). Mild to moderate participants were all non-hospitalized cases. Exclusion criteria were recovery from severe COVID-19 (patients with significant respiratory symptoms and rapid progression on imaging within 24–48 h who need to be hospitalized) and the presence of any cognitive, neurological, metabolic, or orthopedic impairment before COVID-19. The control group included healthy participants with no history of COVID-19 whose age, sex, and physical activity level were matched with the post-COVID-19 group. All participants signed the informed consent form.

### 2.3 Outcome measurements

Participants completed an in-person assessment session that started with recording demographic and clinical characteristics of participants, including age, sex, height, body mass index (BMI), physical activity level, years of education, severity of COVID-19, and time of COVID-19 diagnosis. Persian version of the International Physical Activity Questionnaire Short-Form (IPAQ- SF) was used to determine physical activity level. IPAQ-SF consists of 7 items that assess the frequency and duration of four specific activities, including vigorous-intensity activities, moderate-intensity activities, walking, and sitting for the last 1 week (15, 16). Accordingly, physical activity level was classified as low (<600 MET minutes a week), moderate (600–1,500 min a week), and high (> 1,500 MET minutes a week) (15). In addition, the following measurements with a random order were performed to evaluate physical and cognitive functions and the quality of life. All measurements were performed by the same physiotherapist.

#### 2.3.1 Physical assessments

*Muscle strength* was assessed using the Medical Research Council Sum Score (MRC-SS), that evaluates the strength of six muscle groups, including shoulder abductor, forearm flexor, wrist extensor, hip flexor, knee extensor, and ankle dorsi flexor bilaterally. Each muscle group receives a score between 0 and 5 (0: no visible/palpable contraction; 1: visible/palpable contraction without movement of the limb; 2: movement of the limb but not against gravity; 3: movement against gravity but not against resistance; 4: movement against gravity and resistance; 5: normal), which provides a maximum total score of 60 (17).

*Endurance* was evaluated by the 2 min Step Test. This test requires the participants to march in place as fast as possible while lifting the knees to a height halfway between their patella and iliac crest. The number of right-side steps that reach the criterion height during marching in place for 2 min was recorded (18).

*Fatigue* was determined with the Modified Borg scale at the end of the 2 min Step Test. The participants were asked to rank their perceived level of fatigue from 0 (not fatigued at all) to 10 (total fatigue and exhaustion) (19).

*Physical performance* was assessed using the Short Physical Performance Battery (SPPB). The SPPB has 3 components, including standing balance, 4-meter gait speed, and 5-repetition sit-to-stand tests. Each component is scored from 0 to 4 based on the participant's performance, with the total score ranging from 0 to 12. Scores between 0 and 3 indicate severe physical function disability, 4 to 6 low function, 7 to 9 intermediate function, and 10 to 12 normal function (20, 21).

#### 2.3.2 Cognitive assessments

*Montreal Cognitive Assessment (MoCA)* is a brief global cognitive screening tool that detects mild cognitive impairment by assessing 6 cognitive domains, including executive functions; visuospatial abilities; short-term memory; language; attention, concentration, and working memory; and temporal and spatial orientation. The maximal score is 30 (22, 23).

*Cognitive Failures Questionnaire,* that consists of 25 items, was used to assess subjective cognitive functions (13). Participants are asked to determine how often they make mistakes on a five-point Likert scale, ranging from 0 (never) to 4 (very often), with the highest possible score of 100 (24, 25).

*Trail Making Test (TMT)* consists of TMT-A and TMT-B. In TMT-A that evaluates simple attention, visual scanning, and information processing speed, the participants were asked to connect numbered circles in a numerical order (i.e., 1–2–3, etc.). In the TMT-B that assesses complex attention, working memory, and executive function, participants were required to connect the circles containing numbers and letters in an alternating numeric and alphabetic sequence (e.g., 1-A–2-B). The time to complete each test as fast as possible was measured (26, 27).

*The Stroop color and word test* was used to assess the ability to inhibit cognitive interference that occurred during the simultaneous processing of different features (i.e., ink or word) of one stimulus. Participants were required to press the key with a similar color ink instead of determining the word as quickly as possible (28). Reaction time (Stroop time) and the number of correct answers (Stroop score) were recorded.

*The 5 Dimension 3 Level Euro Quality of Life Questionnaire (EQ-5D-3l)* was used to determine health-related quality of Life. The participants were asked to indicate their health state by choosing the most appropriate answer on a three-level scale (1: no problems, 2: some problems, and 3: extreme problems) in each of the 5 dimensions (mobility, self-care, usual activities, pain/discomfort, and anxiety/depression). In addition, they had to rate their health state on a vertical visual analog scale (EQ-VAS) where the endpoints are labeled “worst imaginable health state” and “best imaginable health state” (29). In this study, the groups were only compared with the EQ-VAS.

### 2.4 Statistical analysis

Data were analyzed using SPSS version 28.0 (IBM Corp, Armonk, New York, USA) with the statistical significance level set at 0.05. Continuous variables are reported as mean and standard deviation or quartile and range, while categorical variables are reported as numbers and percentages. Normal distribution of variables was evaluated by the Shapiro-Wilk test and Q-Q plots. To determine between-group differences, the student's *t*-test or Mann–Whitney *U* test was used for continuous variables, and the Chi-square test was used for categorical variables.

The association between physical and cognitive functions and the quality of life was investigated using Spearman correlation analysis. Correlation was interpreted as very high (*r* = 0.90 to 1), high (*r* = 0.70 to 0.89), moderate (*r* = 0.50 to 0.69), low (*r* = 0.30 to 0.49), and negligible (lower than 0.30) (30).

The sample size was determined based on the data from a pilot study on 20 samples (10 in each group). Using the effect size of 0.8, an alpha level of 0.05, and a power of 0.80, the estimated sample size was 42 (21 in each group). To consider the dropouts, 46 participants were finally included.

## 3 Results

In total, twenty-three people who recovered from COVID-19 (8 males and 15 females with a mean age of 45.13 ± 15.32) disease and 23 healthy controls (8 males and 15 females with a mean age of 40.43 ± 15.79) participated in the study. As indicated in Table 1, the groups had no statistically significant differences in demographic characteristics (*p *> 0.05).

**Table 1:** Demographic and clinical characteristics of participants .

	Post-COVID-19	Control	*P*-value
	(*n* = 23)	(*n* = 23)	
Age (year), Mean (SD)	45.13 (15.32)	40.43 (15.79)	0.620
Sex			
Male, No. (%)	8 (34.5)	8 (34.5)	1.000
Female, No. (%)	15 (65.5)	15 (65.5)	
Height (cm), Mean (SD)	165 (6.2)	163 (6.3)	0.828
BMI (kg/m^2^), Mean (SD)	26.61 (4.25)	26.54 (5.43)	0.166
IPAQ-SF			
Low, No. (%)	9 (39.13)	7 (30.43)	0.667
Moderate, No. (%)	11 (47.82)	14 (60.86)	
High, No. (%)	3 (13.04)	2 (8.69)	
Years of education (year), Mean (SD)	13.91 (3.59)	13.43 (4.01)	0.502
Disease severity			
Mild, No. (%)	9 (39.13)	-	-
Moderate, No. (%)	14 (60.87)	-	-
Time since disease (days), Mean (SD)	128.78 (32.97)	-	-

COVID-19, coronavirus-disease 19; BMI, body mass index; IPAQ-SF, international physical activity questionnaire-short form.

The results of the between-group comparison of physical performance measures are presented in Table 2. These results revealed significant between-group differences in the 2 min Step Test (*p *< 0.001), Borg scale score (*p *< 0.001), and SPPB total score (*p *= 0.03), while the MRC-SS showed no statistically significant between-group differences (*p *= 0.055).

**Table 2:** Comparison of physical performance measures .

Variable	Post-COVID-19	Control	*P*-value	*Z*-value	*r* Effect size
	(*n* = 23)	(*n* = 23)			
MRC-SS	60 (54–60)	60 (60–60)	0.055	−1.921	0.28
2 min Step Test	71 (20–88)	102 (90–158)	**<0** **.** **001**	−3.891	0.57
Modified Borg Scale	4 (3–6)	2 (1–3)	**<0** **.** **001**	−4.112	0.6
SPPB Total Score	6 (4–8)	8 (6–10)	**0** **.** **03**	−2.176	0.32

COVID-19, coronavirus-disease 19; MRC-SS, medical research council sum score; SPPB, short physical performance battery.

Values are expressed as median (IQR).

Significant *p*-values are in bold.

With regards to the comparison of cognitive performance and quality of life measures, Table 3 shows significant between-group differences in MoCA (*p *= 0.003), cognitive failures (*p *< 0.001), TMT-A (*p *= 0.013), TMT-B (*p *= 0.016), Stroop time (*p *< 0.001), Stroop score (*p *< 0.001), and EQ-VAS (*p *= 0.027).

**Table 3:** Comparison of cognitive performance and quality of life measures .

Variable	Post-COVID-19	Control	*P*-value	*Z*-value	*r* Effect size
	(*n* = 23)	(*n* = 23)			
MoCA	27 (14–27)	30 (28–30)	**0** **.** **003**	−3.0	0.44
Cognitive Failures	28 (14–29)	30 (28–30)	**<0** **.** **001**	−3.291	0.48
TMT-A	89.75 (68.14–158.7)	70.66 (60.58–81.45)	**0** **.** **013**	−2.472	0.36
TMT-B	126.18 (96.05–199.01)	95.69 (85.31–132)	**0** **.** **016**	−2.406	0.35
Stroop Time	205.55 (155.61–380.42)	130.57 (107.24–152.16)	**<0** **.** **001**	−4.163	0.61
Stroop Score	74 (67–75)	78 (77–80)	**<0** **.** **001**	−5.152	0.75
EQ-VAS	75 (60–85)	80 (75–90)	**0** **.** **027**	−2.217	0.32

COVID-19, coronavirus-disease 19; MoCA, montreal cognitive assessment; TMT, trail making test; EQ-VAS, EuroQol-visual analogue scales.

Values are median (range).

Significant *p*-values are in bold.

Associations between physical and cognitive functions and the quality of life in post-COVID-19 individuals are presented in Table 4. All physical and cognitive function measures (except Stroop score) had a significant moderate to high correlation with the quality of life.

**Table 4:** Associations between physical and cognitive functions and the quality of life in post-COVID-19 patients (*n* = 23).

Variable	EQ-VAS	
	Correlation Coefficient	*P*-value
Physical Functions		
MRC-SS	0.680	**<0** **.** **001**
2 min Step Test	0.707	**<0** **.** **001**
Modified Borg Scale	−0.566	**0** **.** **005**
SPPB	0.748	**<0** **.** **001**
Cognitive Functions		
MoCA	0.601	**0** **.** **002**
Cognitive Failures	0.809	**<0** **.** **001**
TMT-A	−0.792	**0** **.** **001**
TMT-B	−0.750	**<0** **.** **001**
Stroop Time	−0.555	**0** **.** **006**
Stroop Score	−0.134	0.542

COVID-19: coronavirus-disease 19; MRC-SS: medical research council sum score; SPPB: short physical performance battery; EQ-VAS, EuroQol-visual analogue scales; MoCA: montreal cognitive assessment; TMT: trail making test.

Significant *p*-values are in bold.

## 4 Discussion

The results of the present study demonstrated significant impairments in physical functions in terms of endurance, fatigue, and physical performance among post-COVID-19 participants compared to healthy controls. In addition, cognitive functions, including global cognitive skills, subjective cognitive functions, information processing, executive function, and inhibitory control, and the quality of life were significantly impaired in post-COVID-19 individuals compared to controls. However, there were no between-group differences in muscle strength. Moreover, the results indicated significant moderate to high correlations between physical and cognitive functions and the quality of life in post-COVID-19 individuals.

We observed reduced muscle endurance in post-COVID-19 adults on average 4 months after symptomatic COVID-19, while physical strength was unaffected. Impaired muscle endurance can be the result of decreased maximal aerobic capacity, which has been indicated by lower VO2 max following severe acute respiratory syndrome coronavirus 2 (SARS-CoV-2) infection (31, 32). Reduced muscle endurance can increase fatiguability during the activity of daily living and decrease functional performance, which was also confirmed in our study. Studies with mostly non-hospitalized post-COVID-19 participants reported fatigue in 82% (4), slight to moderate functional limitations (self-reported) in 94% (33), and handgrip and quadriceps weakness assessed by dynamometer in 39.6% and 35.4%, respectively (21) at least 3 months after acute COVID-19. Recently, Gunnarsson et al. reported the prevalence of physical impairments in non-hospitalized post-COVID-19 participants as 20% in functional exercise capacity (assessed by the 6 min Walk Test) and 59% in lower extremity muscle strength and function (30 s Sit-to-Stand Test), and 33% in handgrip strength 3 months following acute illness (7). In the present study, MRC-SS was used to assess muscle strength. Although this is a feasible clinical tool, there is the possibility of showing a ceiling effect, especially in people with higher physical function, and so limits the ability to detect minimal impairment in muscle strength (34).

Another finding of the present study was impaired cognitive functions in post-COVID-19 participants compared to controls. We determined these impairments in both the objective and subjective measures of cognition. Consistent with our study, in a study on mild to moderate severity COVID-19 survivors, 40% of participants showed cognitive impairments, especially in executive function measured by the Stroop test. However, their assessment was via videoconference, and acute COVID-19 patients (lower than 1 month) were also included (35). In addition, Miskowiak et al. reported impairments in different cognitive domains, including general cognitive function and executive function measure by TMT-B, among patients in a long-COVID clinic on average 7 months after acute COVID-19 compared to healthy controls (13). This study included hospitalized and non-hospitalized post-COVID-19 participants with pre-existing respiratory disease such as asthma, and was conducted before the Omicron peak. In another study, Gunnarsson et al. reported lower performance in general cognitive function in hospitalized post-COVID-19 adults compared to the non-hospitalized group, but the TMT-B was similar across the groups (7). The mean TMT-B in our study is higher than the non-hospitalized group in the study by Gunnarsson et al. (154.67 (71.25) seconds vs. 88.8 (44.5)) (7) that may be explained by the lower educational level of participants in our study. Also, Akinci et al. showed worse performance on MoCA and TMT-A tests in young post-COVID-19 adults with mild to moderate disease 21 to 60 days following acute illness compared to healthy controls; however, there were no significant between-group differences in attention span, Stroop time, Stroop Error, and TMT-B tests (36). Moreover, Kirchberger et al., reported that 55.6% of non-hospitalized post-COVID-19 participants 9 months after acute infection had at least mild cognitive impairments in one cognitive test, however, the Stroop test showed deteriorated performance in only 3% of participants (28). Although there is heterogeneity in methodology, executive function is mentioned as the most affected cognitive domains in post-COVID-19 studies (7, 13, 35, 36). Considering the critical role of executive function in in daily living activities and participation, this impairment can negatively affect personal and social life. The mechanism of how COVID-19 leads to cognitive impairment has not been completely proven yet. However, some studies have shown that SARS-CoV-2 can damage the CNS in different ways including direct infection, viruses entering through blood circulation and neuronal pathways, hypoxic and immune injury, and also binding to the angiotensin-converting enzyme 2 (ACE2) receptor, ultimately producing acute and long-term neurological effects (37). Furthermore, as the olfaction and cognitive functions share common neurocircuitry in the frontal lobe, the high incidence of anosmia following COVID-19 infection, which is also one of the most common persistent symptoms in post-COVID-19 cases, draws attention to the role of olfactory neuroinflammation in cognitive changes, especially executive function (35, 38).

Interestingly, both the persistent physical and cognitive impairments in post-COVID-19 participants had significant moderate to high correlations with the health-related quality of life. Consistent with our findings, Miskowiak et al. found correlation between subjective and objective cognitive function and quality of life 7 months after COVID-19 (13). Quality of life is a subjective concept that includes physical and mental health. Affects of COVID-19 on the quality of life can place higher demands on the healthcare system in the long term. The association of quality of life and physical and cognitive function should be considered for designing health-promoting strategies post-COVID-19 population.

### 4.1 Strengths and limitations

Several studies have reported the high prevalence of physical and cognitive impairments in post-COVID-19 individuals (2, 6, 11, 21, 39). However, most of these studies were performed on hospitalized patients, focused on patient-reported symptoms, included people with predetermined medical conditions (e.g., diabetes and cancer), and did not compare the results with a control group. In the present study, we included non-hospitalized post-COVID-19 participants without pre-existing locomotor disabilities and baseline conditions and compared the results with a control group.

This study acknowledges some limitations. First, all the participants of the present study had mild to moderate disease severity without the need for hospitalization, so we would urge caution on generalizing the findings to those with post-COVID-19 who had been hospitalized. However, we suggest that our assessment of physical and cognitive function in people with post-COVID-19 who were not hospitalized and did not have pre-existing medical conditions represents the novelty of our findings. Second, we did not use any objective biomarker-based measure to indicate whether a COVID-19 infection has occurred in controls. Third, the study participants were infected during the peak of the SARS-CoV-2 Omicron variant, and the results may not be transferrable to later virus variants. Fourth, muscle strength was assessed with MRC-SS which may exposed to ceiling effect. It is recommended that future studies use a more sensitive tool to assess muscle strength. In addition, a regression analysis on a larger sample can provide a better understanding of the impact of COVID-19 on physical and cognitive function.

## 5 Conclusion

Overall, the results showed that post-COVID-19 participants with mild to moderate disease severity encountered significant impairments in physical and cognitive function, which were correlated with impaired quality of life. These findings can highlight the need for a compressive assessment to detect impairments across multiple domains and plan the appropriate management strategies aiming at health promotion.

## Data Availability

The raw data supporting the conclusions of this article will be made available by the authors, without undue reservation.
